# Clinicians’ attitude towards family planning and timing of diagnosis in autosomal dominant polycystic kidney disease

**DOI:** 10.1371/journal.pone.0185779

**Published:** 2017-09-29

**Authors:** Stéphanie De Rechter, Jonathan Kringen, Peter Janssens, Max Christoph Liebau, Koenraad Devriendt, Elena Levtchenko, Carsten Bergmann, François Jouret, Bert Bammens, Pascal Borry, Franz Schaefer, Djalila Mekahli

**Affiliations:** 1 Department of Pediatric Nephrology, University Hospital of Leuven, Leuven, Belgium; 2 Department of Development and Regeneration, KU Leuven, Leuven, Belgium; 3 University of New Haven, New Haven, CT, United States of America; 4 Department of Nephrology, University Hospital of Brussels, Brussels, Belgium; 5 Department of Pediatrics and Center for Molecular Medicine, University Hospital of Cologne, Cologne, Germany; 6 Department of Genetics, KU Leuven—University Hospital of Leuven, Leuven, Belgium; 7 Center for Human Genetics, Bioscientia, Ingelheim, Germany; 8 Department of Medicine, University Hospital of Freiburg, Freiburg, Germany; 9 Division of Nephrology, University of Liège Hospital (ULg CHU), Liège, Belgium; 10 Groupe Interdisciplinaire de Génoprotéomique Appliquée (GIGA), Cardiovascular Sciences, University of Liège, Liège, Belgium; 11 Department of Microbiology and Immunology, KU Leuven, Leuven, Belgium; 12 Department of Nephrology, Dialysis and Renal Transplantation, University Hospital of Leuven, Leuven, Belgium; 13 Centre for Biomedical Ethics and Law, Department of Public Health and Primary Care, University of Leuven, Leuven, Belgium; 14 Division of Pediatric Nephrology, Centre for Pediatrics and Adolescent Medicine, Heidelberg University Medical Centre, Heidelberg, Germany; Istituto Di Ricerche Farmacologiche Mario Negri, ITALY

## Abstract

Several ethical aspects in the management of Autosomal Dominant Polycystic Kidney Disease (ADPKD) are still controversial, including family planning and testing for disease presence in at-risk individuals. We performed an online survey aiming to assess the opinion and current clinical practice of European pediatric and adult nephrologists, as well as geneticists. A total of 410 clinicians (53% male, mean (SD) age of 48 (10) years) responded, including 216 pediatric nephrologists, 151 adult nephrologists, and 43 clinical geneticists. While the 3 groups agreed to encourage clinical testing in asymptomatic ADPKD minors and adults, only geneticists would recommend genetic testing in asymptomatic at-risk adults (*P*<0.001). Statistically significant disagreement between disciplines was observed regarding the ethical justification of prenatal genetic diagnosis, termination of pregnancy and pre-implantation genetic diagnosis (PGD) for ADPKD. Particularly, PGD is ethically justified according to geneticists (4.48 (1.63)), whereas pediatric (3.08 (1.78); *P*<0.001) and adult nephrologists (3.66 (1.88); *P*<0.05) appeared to be less convinced. Our survey suggests that most clinicians support clinical testing of at-risk minors and adults in ADPKD families. However, there is no agreement for genetic testing in asymptomatic offspring and for family planning, including PGD. The present data highlight the need for a consensus among clinicians, to avoid that ADPKD families are being given conflicting information.

## Introduction

Autosomal dominant polycystic kidney disease (ADPKD) is the most common hereditary kidney disease [1, 2]. In the absence of a cure, half of the patients currently develop end stage renal disease (ESRD) in their fifth or sixth decade, requiring renal replacement therapy [3]. Since most patients remain *a-* or *oligo-*symptomatic until adulthood, ADPKD is usually regarded as a late-onset disease [4]. However, evidence is cumulating that renal injury starts early in life, with the formation of renal cysts *in utero* [5]. Moreover, 2–5% of ADPKD patients present in childhood with a broad phenotypic spectrum, ranging from a severe neonatal presentation [6] to the incidental finding of renal cysts detected on ultrasound [7]. Children diagnosed with ADPKD have proteinuria in up to 35% of cases, hypertension before a renal function decline in up to 44% and more than half have urinary concentrating defects [4]. Significant irreversible destruction of renal parenchyma will occur long before clinical symptoms develop or a loss in glomerular filtration rate (GFR) is noted, due to hyperfiltration and hypertrophy of residual nephrons [8].

Whether asymptomatic at-risk individuals should be tested for the presence of the disease is still a matter of controversy [9]. On the one hand, the absence of an effective cure [3], the potential psychological stress related to the diagnosis of a chronic progressive disease in the context of affected family members, possibly causing survival guilt or even ostracism [10, 11], and potential financial and legal implications such as the inability to obtain life or medical insurances [12] have been put forward against pre-symptomatic testing. On the other hand, presymptomatic testing has prognostic implications (*PKD1* versus *PKD2*) and may induce early targeting of modifiable risk factors for disease progression [13], including hypertension [14–16], proteinuria, urological complications [17] and hypercholesterolemia [18], increasing the effectiveness of interventions to improve long-term renal survival [19]. This is indirectly supported by the evidence of slower cyst growth in number and size in normotensive compared to hypertensive children with ADPKD [20]. Effective blood pressure control from childhood on may also improve cardiovascular outcomes in this patient group, at high risk for early cardiovascular events [21, 22]. A benefit accruing to all tested individuals, is the potential for increased control over their own health and life, among other things informed reproductive decision making [11].

ADPKD is most commonly diagnosed based on the family history and sonographic [23, 24] or magnetic resonance imaging (MRI) findings [25]. However, for individuals younger than 15 years, uniform imaging diagnostic criteria are lacking [26],[27]. Moreover, definite exclusion of ADPKD based on imaging is only possible in at-risk individuals aged 30–40 years or older [23–25]. A definite diagnosis based on gene sequencing is not yet routinely used in clinical practice given the presence of six *PKD1* pseudogenes and tremendous allelic heterogeneity, making molecular genetic testing challenging and expensive. While the diagnostic accuracy of *PKD1* and *PKD2* gene screening was found to be lower than the accuracy of ultrasound examination in adults beyond the age of 30 years, the relative accuracy of genetic *vs*. ultrasound screening was similar for children younger than 15 years in *PKD1* and superior in *PKD2* individuals [23]. A third diagnostic option is regular monitoring for disease manifestations such as hypertension and proteinuria [4].

The advent of genetic testing for ADPKD [28] and advanced obstetric techniques in assisted reproduction have given rise to new possibilities for prenatal diagnosis [29, 30] and potentially, termination of pregnancy, and for pre-implantation genetic diagnosis (PGD) [31]. However, the availability and financial coverage of these techniques varies from country to country [19]. Moreover, although evolving, the European legal landscape regarding practices in genetics, PGD, and the governmental policies on the use of genetic information in insurance and employment is still very heterogeneous. Some countries have comprehensive provisions pertaining to genetic testing in their biomedical and bioethical regulation (Norway [32], Spain [33] and France [34]), while others have enacted laws specific to genetics [35] (Austria [36], Germany [37], Hungary [38], Portugal [39], Sweden [40] and Switzerland [35]) or address genetics within more general laws on health care issues (Czech Republic [41], Ireland [42] and Lithuania [43]). In countries where genetic testing is not regulated by specific laws or provisions, regulation related to patient rights and health care professionals’ duties is applied [44], e.g. in Belgium [45]. The same applies to assisted reproduction: according to Turillazzi et al. and Harper et al., PGD is outright banned in Austria and Switzerland, while laws in Germany, Ireland and Italy leave some room for interpretation. PGD is allowed in Belgium, Bulgaria, Cyprus, Czech Republic, Denmark, Finland, France, Greece, Latvia, Portugal, Romania, Spain, Sweden and UK [46]; although the allowed indications for PGD vary by country to a major extent [47].

Widely varying opinions towards presymptomatic predictive testing, genetic counseling and family planning for ADPKD have been voiced by patients [48–50]. However, the attitudes of clinicians and the underlying arguments towards these topics have never been studied; only the opinion of nephrologists on screening modalities for unruptured intracranial aneurysms in ADPKD has been published recently [51].

Therefore, the aim of this study was to gather representative information on these ethical issues, from European pediatric and adult nephrologists and clinical geneticists.

## Subjects and methods

### Procedure

An online questionnaire was designed by 7 experts in the field of ADPKD, including 2 pediatric and 3 adult nephrologists, as well as 2 clinical geneticists. The design was then validated and approved by a group of 8 other ADPKD experts (3 pediatric, 3 adult nephrologists and 2 geneticists). After approval by the Ethics Committee of Leuven University, the project was approved and endorsed by the Working Group for Inherited Kidney Disorders of the European Society of Pediatric Nephrology. To avoid sample bias, we chose to contact pediatric nephrologists and geneticists throughout Europe. As this approach was not feasible for adult nephrologists, we mainly focused on Germany and Belgium as two exemplary European countries differing in legislation regarding genetic testing, as this might affect the clinicians’ clinical practice and opinions. Belgium has no specific legislation on genetic testing [35], while Germany has an elaborated and specific legal framework [52]. PGD has only become legal in Germany in December 2011 and is restricted to cases where the parents have a predisposition to a serious genetic illness [53], as an exception to the Embryo Protection Act which banned PGD in 1990 [54].

To contact adult nephrologists, we used the national mailing lists from Belgium and Germany. Pediatric nephrologists were contacted via the ESPN membership mailing list, consisting of 1938 recipients, including fellows and non-European members. Geneticists were recruited via the Facebook and Twitter groups of the European Society for Human Genetics and via a mailing list, obtained as previously described [55]. Two reminders were sent out at 2-week intervals. No monetary or other incentive was offered to the caregivers. We complied with the terms of service for the website from which we collected the data for analysis.

### Questionnaire

A 17-item questionnaire was developed for pediatric and adult nephrologists, and an adapted 15-item questionnaire for geneticists. The survey instruments (Supporting information S1 and S2 Appendices) included 3 sections.

First, sociodemographic factors including gender, age, country, and practice/center characteristics were collected. Countries were divided in 4 groups based on geographical regions described by the United Nations [56].

Second, multiple-choice questions aimed at defining the clinicians’ current clinical practice. We assessed (i) whom the respondents consider responsible for informing the minor about his/her genetic risk, (ii) which methods they apply for testing asymptomatic at-risk individuals, and (iii) whether they inform their patients about the possibilities of prenatal diagnostics and PGD. Next, their recommendation was asked for the management of a fictitious case: “a 35-year-old ADPKD patient with an asymptomatic child of 6 years, and several affected family members are known with early disease manifestation”.

Third, a series of statements were used to establish the clinicians’ opinions and beliefs regarding clinical and ethical issues such as predictive (genetic) testing in at-risk individuals, prenatal genetic diagnosis by means of chorionic villus sampling or amniocentesis, termination of pregnancy and PGD for ADPKD. We used a 6-point Likert response scale, in which a score of 1 means the respondent strongly disagrees, and a score of 6 means a strong agreement on a given statement. In the *Results* section answers are shown as the mean of the numerical mean score and standard deviation (SD). At-risk individuals were defined as first-degree relatives of individuals diagnosed or suspected to have ADPKD. Testing for ADPKD comorbidities such as hypertension and proteinuria was considered as clinical testing.

### Statistical analysis

Statistical analyses were performed using Stata 14/SE. Given that individual survey questions collected data in a variety of ways (e.g., dichotomous nominal, multi-level nominal and ordinal, and six-point Likert scales), different types of analysis were performed to assess various questions. Relationships between categorical responses were assessed using Chi-square. Six-point Likert data measuring agreement with prompts were treated as numeric allowing t-tests to be utilized for group comparisons. For all analyses a 0.05 significance level was used for establishing statistical significance. However, the Bonferroni correction was employed to control for type I error inflation through multiple comparisons. Missing data were handled through list-wise deletion for each specific test. Dichotomous responses on who is responsible for informing at-risk individuals were recoded into a five-point ordinal scale (only parents, mainly parents, parents & professionals, mainly professionals, only professionals). These data were utilized to generate figures for graphical analysis only. For the current approach on informing on both prenatal genetic diagnosis and PGD, logistic regression analysis was performed to detect the possible influence of gender, work setting, ADPKD research involvement, the availability to genetic testing and/or counseling and possible reasons not to test their patients for disease presence such as inducing stress, financial implications, the absence of a curative treatment.

Finally, to determine the impact of legislative differences in Germany, Ordinary Least Squares (OLS) regression models were estimated using the six-point Likert items indicating agreement as dependent variables. Models were verified through Independent and Identically Distributed (IID) testing, which indicated no model bias due to the nature of the dependent variables.

## Results

### Study population

A total of 410 physicians completed the online survey. For pediatric nephrologists, 686 out of 1938 recipients (35.4%) opened the mail, of whom 216 (31.5%) responded on the questionnaire. Their characteristics are given in Table 1. Most of the respondents work in an academic setting and one third was or is involved in patient-oriented ADPKD research in the past or at present.

**Table 1:** Demographic characteristics of respondents.

	Total Sample (N = 410)	Adult nephrologists (N = 151)	Pediatric nephrologists (N = 216)	Geneticists (N = 43)
Male	53.4%	65.6%	43.1%	62.8%
Mean age (SD)	48.3 (9.8)	46.9 (10.2)	48.3 (9.3)	52.8 (9.9)
Country				
**1.** *Western Europe*	55.4%	90.1%	34.7%	37.2%
○ *Belgium*	28.1%	60.9%	8.8%	9.3%
○ *Germany*	15.4%	23.2%	10.2%	14.0%
*2. Eastern Europe*	6.6%	2.0%	8.3%	14.0%
*3. Northern Europe*	12.4%	0.7%	17.6%	27.9%
*4. Southern Europe*	16.3%	6.6%	22.2%	20.9%
*5. Others*	9.3%	0.7%	17.1%	0.0%
Academic work setting	70.5%	42.4%	86.6%	88.4%
Involved in ADPKD research	32.9%	31.8%	33.8%	32.6%
Access to genetic testing for ADPKD	56.8%	49.7%	58.3%	74.4%
Access to genetic counseling for ADPKD patients	79.8%	70.2%	82.4%	100%

### Current clinical practice

**Fig 1:**
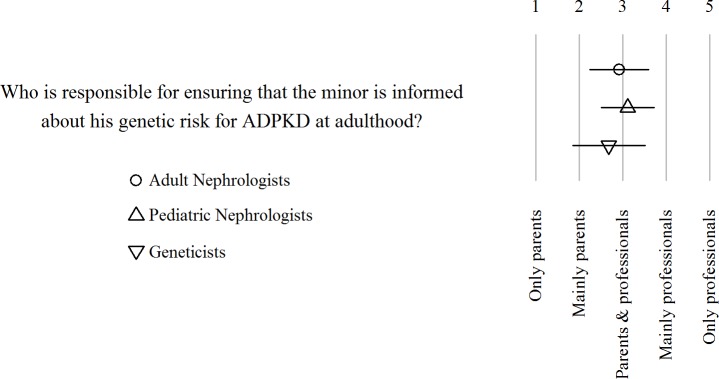
Responsibility of informing at-risk individuals about their genetic risk. Dot, triangles and square represent mean per group, lines ± 1 SD.

All respondents agreed that the task of informing an at-risk minor about his or her genetic risk for ADPKD at adult age should be a shared responsibility of the professional care givers and the parents (Fig 1).

**Fig 2:**
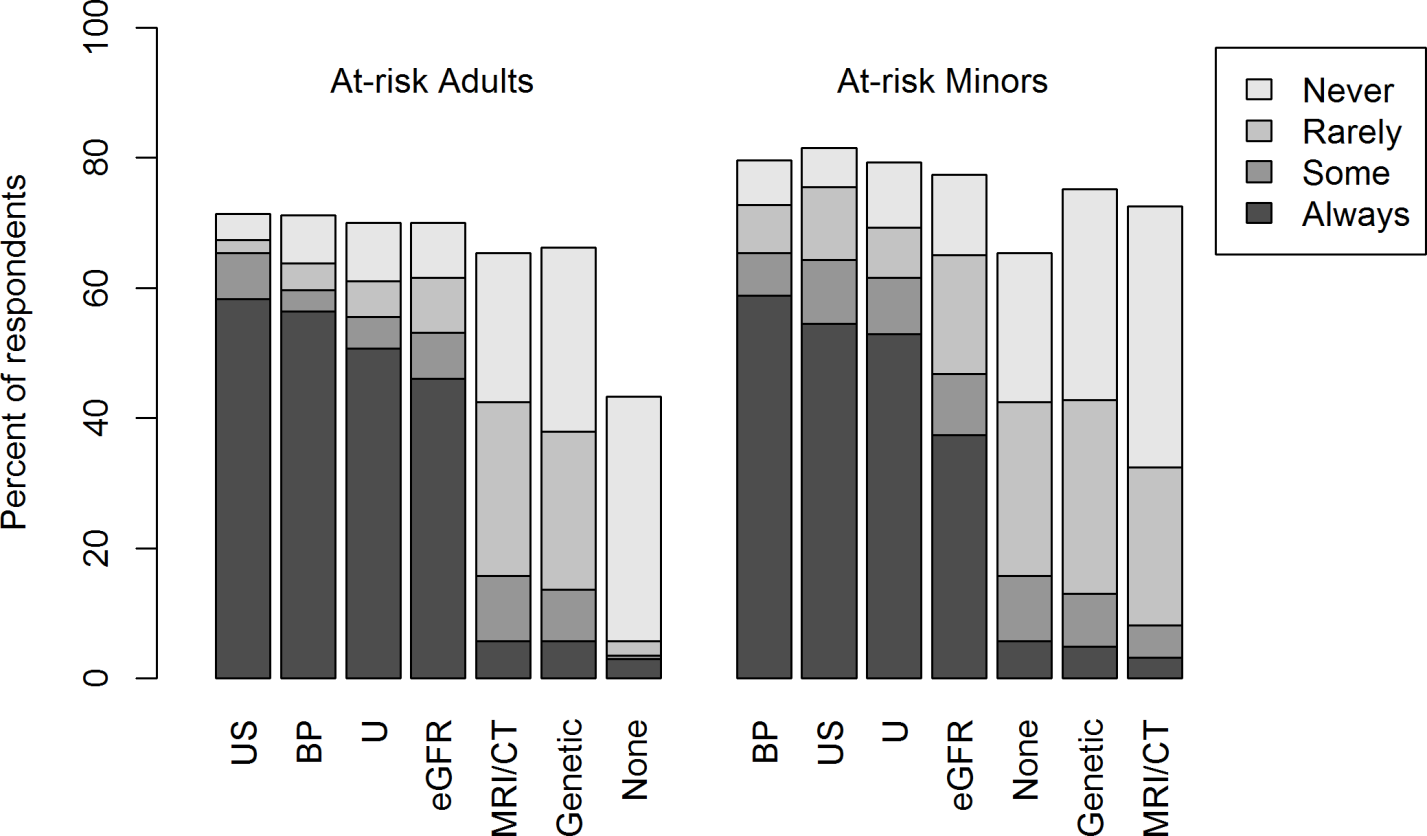
Use of different diagnostic techniques to test for ADPKD in at-risk adults and minors. Abbreviations: BP: blood pressure, eGFR: estimated glomerular filtration rate, U: urine analysis, US: ultrasound, MRI/CT: magnetic resonance imaging / computed tomography, Genetic: genetic testing, None: none of the previously mentioned techniques.

Considering the diagnostic methods applied to test for the presence of ADPKD in asymptomatic at-risk individuals, ultrasound, blood pressure measurement and urinalysis are routinely used by 50–60% of both adult and pediatric nephrologists (Fig 2). GFR is estimated by 50% of adult but only 35% of pediatric nephrologists. Most adult and pediatric nephrologists use rarely MRI, computed tomography (CT) and genetic screening, unless in selected patients.

**Fig 3:**
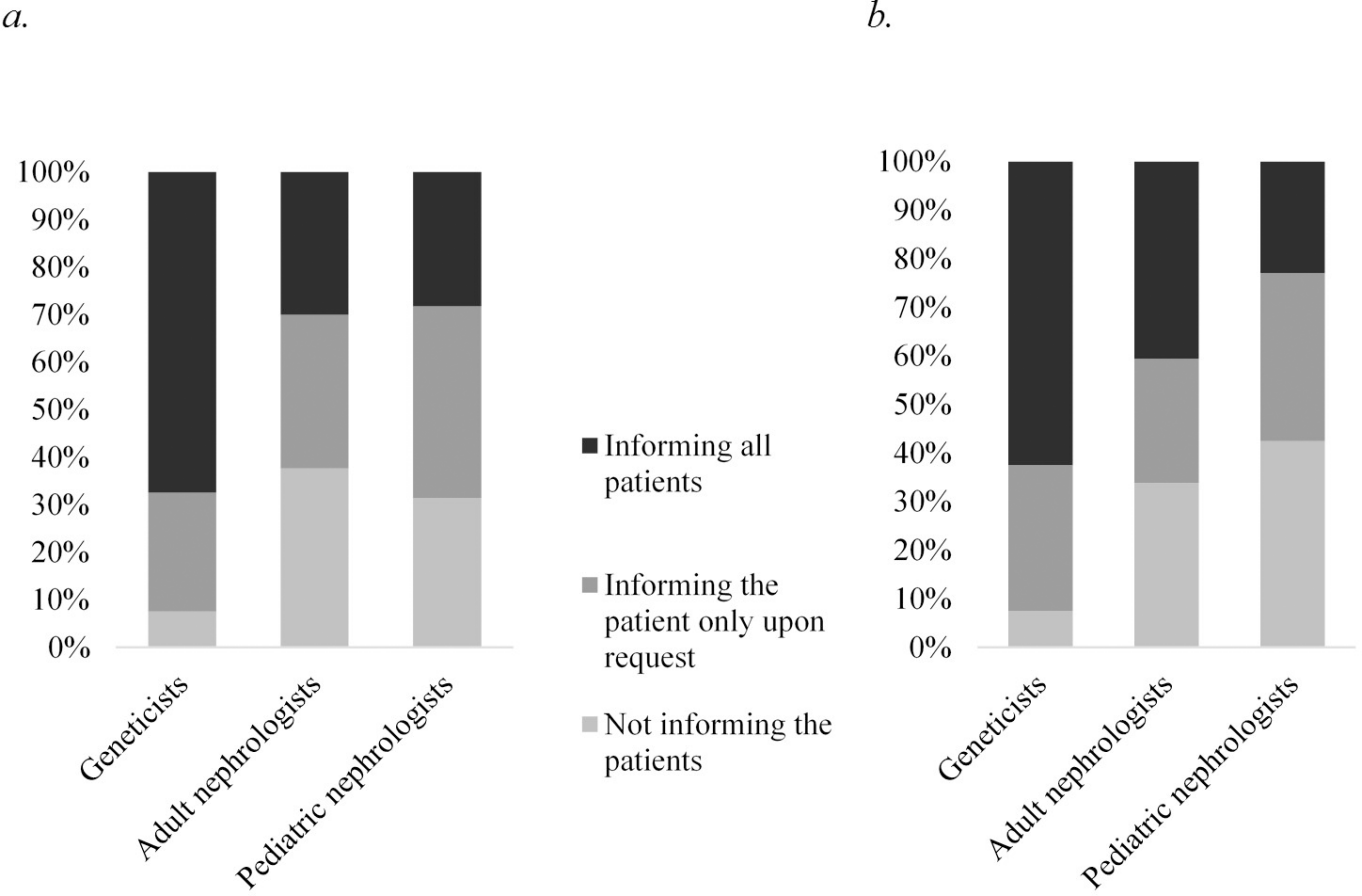
Current practice on informing the patient about the possibility of (a) prenatal genetic diagnosis by chorionic villus sampling or amniocentesis and (b) pre-implantation genetic diagnosis (PGD) for ADPKD.

The attitude towards informing ADPKD patients about the possibility of prenatal genetic diagnosis by means of chorionic villus sampling or amniocentesis, in case of future pregnancies, differed distinctly between the disciplines (*P*<0.001). Two thirds of geneticists actively inform the families, the other third does this upon patient request. In contrast, only a minority of adult and pediatric nephrologists routinely informs their patients (Fig 3A). Likewise, geneticists almost unanimously inform their patients about the possibility of *in vitro* fertilization with PGD either routinely (63%) or upon request (30%), whereas only 41% of adult and 23% of pediatric nephrologists inform their patients about this option (*P*<0.001) (Fig 3B). The differences in counseling attitude were not attributable to any factors other than the professional discipline.

Current clinical practices were evaluated based on a fictitious clinical case of an asymptomatic child with a family history of ADPKD. Of the 410 respondents, an annual blood pressure measurement and urine checkup was recommended by 251 respondents (61.2%). Eighty (19.5%) recommended genetic testing for both parent and child and 73 (17.8%) recommended clinical and/or genetic testing only when the child would have reached adult age. Hundred fifty two (37.1%) respondents advised against investigations in childhood. Of these, 24 (15.8%) argued that the disease does not manifest before adulthood, 47 (30.9%) justified postponing diagnostic evaluation by the risk of inducing psychological stress, 41 (27%) cited the current unavailability of an efficacious treatment, 33 (21.7%) potential insurance problems, and 7 (4.6%) reasoned that no definite diagnostic method exists at childhood age. Geneticists were significantly more likely to recommend genetic testing at adult age than nephrologists (*P*<0.001).

### Views on controversies and ethical issues

#### Clinical and predictive genetic testing in at-risk individuals

**Fig 4:**
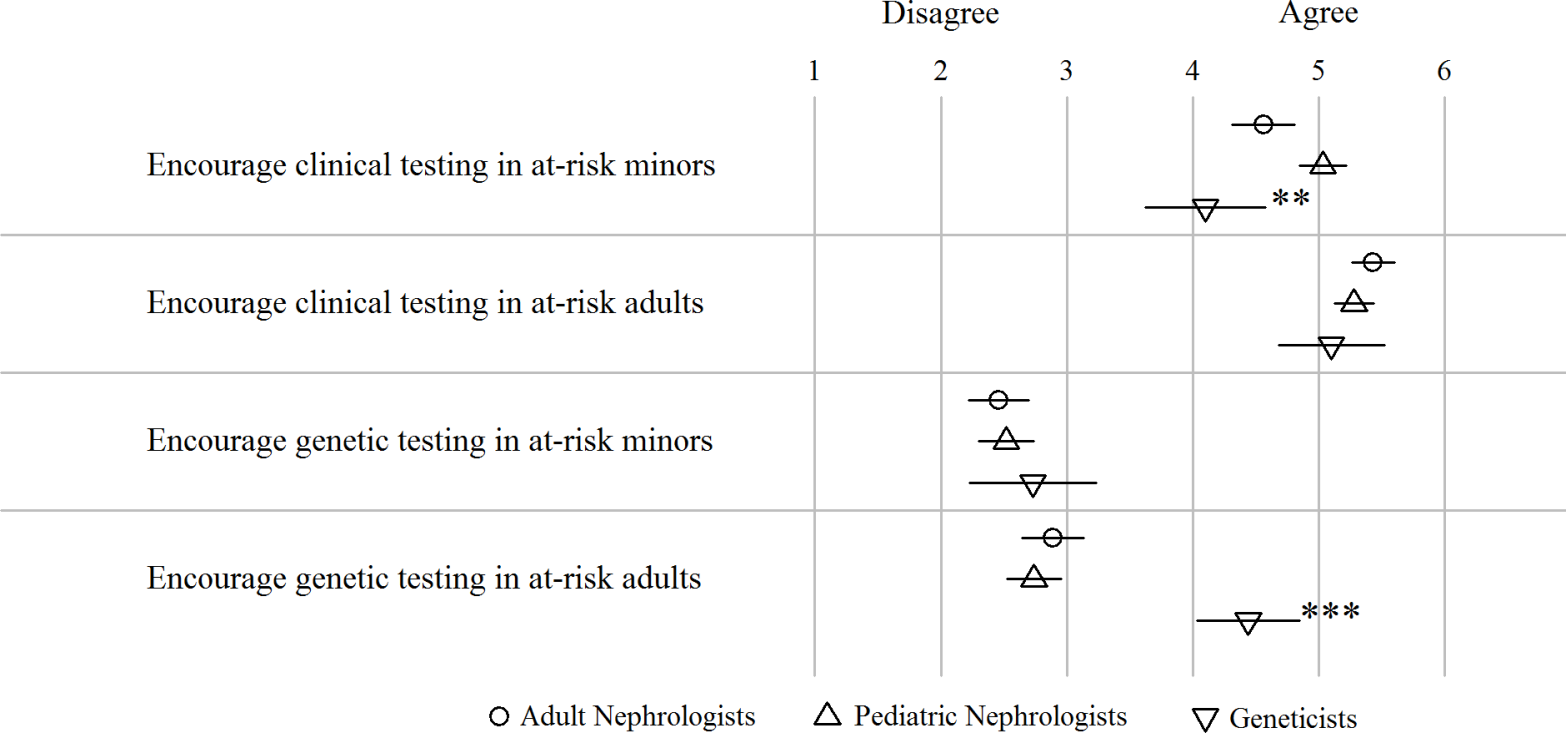
(Dis)agreement on proposing clinical and genetic testing in at-risk minors and adults. Scoring ranged from 1 = strongly disagree to 6 = strongly agree. Dots, triangles and squares represent mean per group, lines the 95% confidence interval. **: *P*<0.01, ***: *P*<0.001, for difference between geneticists and combined nephrologist groups.

The three groups of clinicians similarly supported clinical testing in at-risk adults (full sample mean (SD): 5.31 (1.16) points on a 6-point scale) (Fig 4). Older clinicians were less likely to agree on this (*P*<0.05).

While all groups encouraged clinical testing in at-risk minors (full sample mean (SD): 4.76 (1.50)), pediatric nephrologists showed significantly stronger support for seeking a diagnosis on a clinical basis in children at-risk compared to geneticists (*P*<0.001). In the multivariate analysis, adult nephrologists working in an academic setting were more supportive of clinical testing of minors (*P*<0.01). Those unwilling to test for the presence of the disease because of the perceived absence of curative treatment options or because they considered the disease as manifesting only in adulthood, were less likely to agree with clinical testing of minors (*P*<0.05 and *P*<0.01, respectively).

All groups moderately disagreed on performing genetic testing in at-risk minors (full sample mean (SD): 2.53 (1.57)). Genetic testing in at-risk adults was favored by geneticists but not by the nephrologist groups (*P*<0.001). Clinicians working in Germany were less in favor of genetic testing compared to clinicians working elsewhere: mean (SD) for at-risk minors was 1.83 (1.49) for clinicians working in Germany. For clinicians elsewhere, this was 2.30 (1.71) (*P*<0.01). For at-risk adults, mean (SD) was 2.27 (1.63) for clinicians working in Germany compared to 2.65 (1.84) for clinicians working elsewhere (*P*<0.05).

#### Family planning in ADPKD

**Fig 5:**
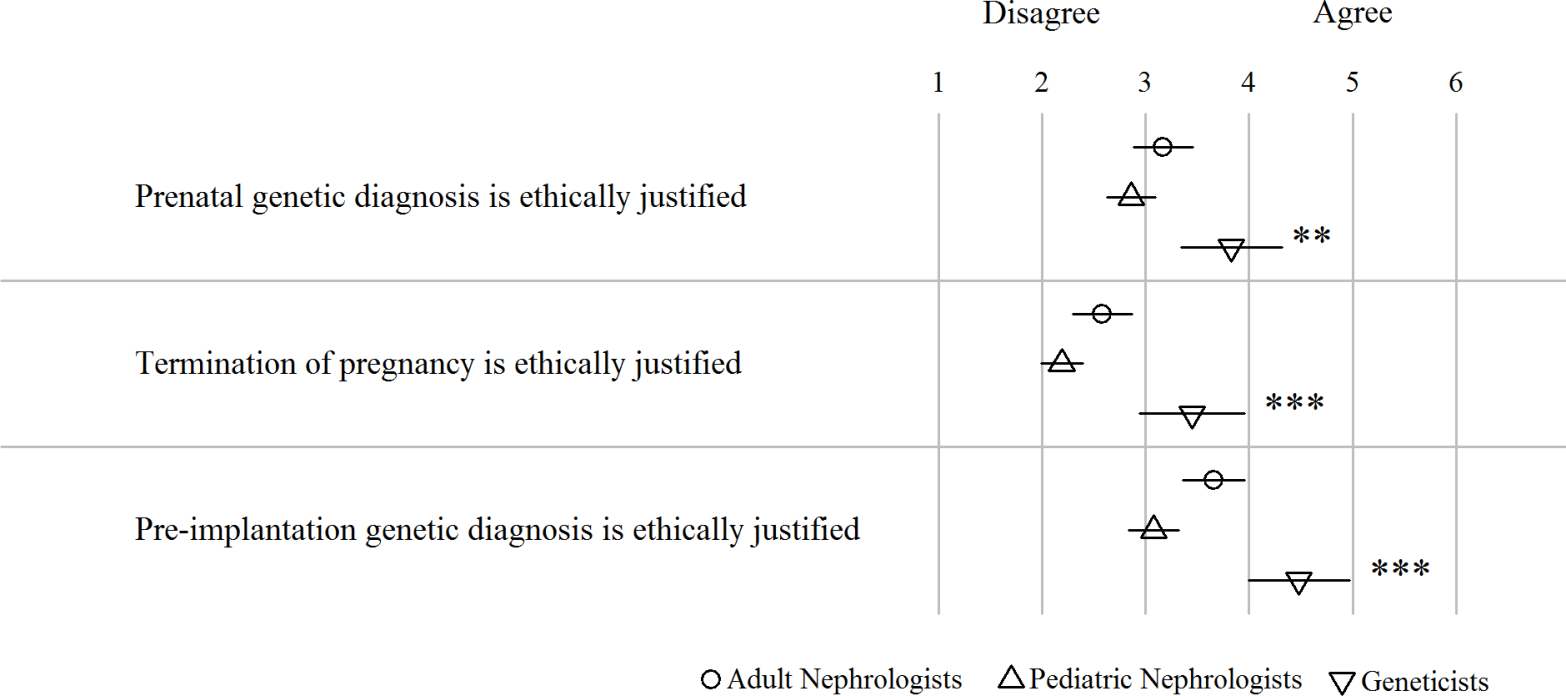
(Dis)agreement on ethical justification of prenatal diagnostics, termination of pregnancy and preimplantation genetic diagnostics (PGD) in pregnant women with ADPKD. Scoring ranged from 1 = strongly disagree to 6 = strongly agree. Dots, triangles and squares represent mean per group, lines the 95% confidence interval. **: *P*<0.01, ***: *P*<0.001, for difference between geneticists and combined nephrologist groups.

The respondents tended to disagree with the statement that prenatal genetic diagnostics, by means of chorionic villus sampling or amniocentesis, is ethically justified (full sample mean (SD): 3.08 (1.76)) (Fig 5). Moreover, adult nephrologists working in an academic setting, and German respondents were more likely to disagree (*P*<0.01 and *P*<0.001, respectively). However, geneticists exhibited a more positive view on prenatal genetic testing compared to pediatric nephrologists (*P*<0.01) and adult nephrologists (*P*<0.05).

The respondents also disagreed with the statement that termination of pregnancy for fetuses diagnosed with ADPKD is ethically justified (full sample mean (SD): 2.78 (1.67)). However, geneticists had a more liberal view on this issue than pediatric (*P*<0.001) and adult nephrologists (*P*<0.01).

Geneticists strongly felt that PGD is ethically justified if a parent-to-be suffers from ADPKD (4.48 (1.63)), whereas pediatric (*P*<0.001) and adult nephrologists (*P*<0.05) had a rather neutral view on this new technology.

Academic adult nephrologists and respondents from Germany were less likely to agree with either termination of pregnancy or PGD (*P*<0.001 for both).

## Discussion

This study provides an assessment of ethical views and management attitudes among European healthcare professionals towards ADPKD. To date, only three reports on ethical issues in ADPKD have been published, focusing on patients’ opinions [48–50]. Two of them were performed in the nineties [48, 50]. It is important to emphasize that this survey was performed prior to the publication of the recent Kidney Disease: Improving Global Outcomes (KDIGO) consensus, the first initiative to provide clinical practice guidelines on the management of ADPKD [19], the European ADPKD Forum (EAF) Report [57] and prior to the approval of tolvaptan use by the European Medicines Agency (EMA) [58].

The respondents broadly agreed that both minors and adults at-risk for ADPKD should be tested for the presence of ADPKD. Notably, the support for clinical testing of minors was the strongest among pediatric nephrologists, in keeping with their professional focus and greater exposure to patients with early-onset symptomatic disease [4]. KDIGO participants reached consensus that presymptomatic testing is not recommended for at-risk minors, but solely for at-risk adults, by means of ultrasound or MRI. However, the report suggests at-risk children to be screened for hypertension from the age of 5, with intervals of 3 years if screening is negative [19]. The latter is in line with our observation that all disciplines encouraged clinical testing for at-risk minors.

The most common arguments raised against testing of at-risk minors in this study were the induction of psychological stress and the absence of a treatment–at that time—for the disease. At least the latter notion requires re-consideration in the light of the approval of the use of tolvaptan for ADPKD by the EMA and its current use in selected adult patients [58].

The current prioritization of diagnostic measures in ADPKD screening was similar for adults and minors, with ultrasound, blood pressure monitoring and urine analysis being the preferred tools by the majority of respondents. Most adult and pediatric nephrologists appear to choose MRI, CT and genetic testing only in selected cases. While MRI has a higher sensitivity in detecting small renal cysts in comparison with sonography [59], its use is still limited by higher cost and the need for sedation in young children. The respondents clearly exhibited a cautionary position towards predictive genetic testing in asymptomatic at-risk individuals, except for geneticists who favored genetic testing in at-risk adults. In line with this clinical practice, the KDIGO report considered molecular testing only to be required in case of atypical renal imaging findings or clinical course, sporadic cases and reproductive counseling. It remains to be seen whether the massive reduction of cost and time by the recent introduction of next generation panel sequencing in routine genetic diagnostics will change the role of genetic screening in ADPKD testing [28, 60].

We noticed large inter- and intra-discipline variety regarding the perceived appropriateness of prenatal genetic diagnosis and termination of pregnancy, or PGD and the routine practice of transmitting the respective information to ADPKD families.

Adult and pediatric nephrologists expressed cautious views on prenatal testing, mostly rejected pregnancy termination and had a neutral attitude regarding PGD, whereas geneticists felt more positively about prenatal genetic testing and potential pregnancy termination and viewed PGD as clearly justified. In keeping with their ethical concerns, only 20–40% of nephrologists reported proactively informing all their patients about the available prenatal and pre-implantation diagnostic options. This behavior contrasts the KDIGO consensus statement that all ADPKD patients should have reproductive counseling and that PGD should be included in this discussion [19]. In this context, a recent report clearly supports the importance of discussing these issues with the patients and their families [49]. Two thirds of ADPKD patients stated that PGD should be made available to prospective parents with this disease. Moreover, 17% of patients not in ESRD and 18% of patients in ESRD would consider prenatal diagnosis and termination of pregnancy; 50% of non-ESRD and 63% of ESRD patients expressed an intention or wish to access PGD for themselves.

An important observation was that physicians working in Germany, consistently had more negative attitudes regarding the ethical acceptability of predictive genetic testing in at-risk individuals, prenatal genetic diagnostics, termination of pregnancy and PGD than physicians from other countries. We hypothesize that this divergent behavior is due to the difference in legislation between Germany and other European countries.

Our study has some limitations. First, we were unable to calculate the exact response rate for adult nephrologists and geneticists as we used several channels to reach the maximum number of people possible. Second, the optimal manner to explore underlying explanations for attitudes regarding these ethical topics would be to perform a live or telephone open-ended question survey. As this was not feasible, we opted for an online closed-ended survey. Of important note, our questionnaire was first designed and then independently validated by experts in the field of ADPKD, including 5 pediatric nephrologists, 6 adult nephrologists and 4 clinical geneticists *in toto*. Still, a selection bias could not be excluded as our questionnaire was only accessible online and in English. Unanswered questions were dealt with through list-wise deletion on a test by test basis.

We conclude that the heterogeneous attitudes observed in this survey within and across disciplines may cause transmission of conflicting information to patients by different clinicians. Establishing a broad intra- and interdisciplinary consensus—if possible as these are sensitive ethical issues—centered around patients’ needs is urgently required. Based on our results, there is a clear need for standardization of care for ADPKD families. We propose to invoke a consensus finding process of multidisciplinary teams at least per center and if possible at a national level. In this process, not only treatment options and extrarenal complications but also practical implications such as potential impact on work, insurance, lifestyle, family planning, and psychological health should be reflected. Once a consensus is reached and implemented as a guideline to clinical practice, patients will no longer receive conflicting information. Importantly, family planning counseling should be made available to all ADPKD patients at initial diagnosis, including genetic counseling and informing them about the possibility of PGD, as suggested by the EAF 2015 Report [5] and the KDIGO consensus [6]. Moreover, affected parents should be informed about screening options for at-risk children. The ultimate goal is to make patients feel sufficiently informed and empowered to make their own decisions. Checklists for both patients and doctors, for initial diagnostics and follow-up care, as suggested by the KDIGO, could facilitate the provision of standardized care.

As a future perspective, it would be interesting to repeat this questionnaire in several years to evaluate the impact of the KDIGO consensus statement and the availability of tolvaptan and other upcoming treatment options on caregivers’ attitudes. Moreover, a comparison of caregiver and patient opinions, surveyed simultaneously in the same geographic area, might reveal important concerns, given the divergence between caregivers’ views compiled in this study and published patient views [47–49].

## Supporting information

S1 Appendix Questionnaire for adult and pediatric nephrologists. (PDF)

S2 Appendix Questionnaire for geneticists. (PDF)

## Abbreviations

ADPKD: autosomal dominant polycystic kidney disease
(e)GFR: (estimated) glomerular filtration rate
KDIGO: Kidney Disease: Improving Global Outcomes
MRI: magnetic resonance imaging
PGD: pre-implantation genetic diagnosis
