# Supplementary material for: Clinicians’ attitude towards family planning and timing of diagnosis in autosomal dominant polycystic kidney disease
Source: PLoS One. 2017 Sep 29;12(9):e0185779. doi: 10.1371/journal.pone.0185779 (PMC5621697; doi:10.1371/journal.pone.0185779)
Supplement: S2 Appendix — (PDF) [file pone.0185779.s002.pdf]

S2 Appendix: Questionnaire for geneticists

1. What is your gender?

Choose: male, female.

2. What is your age (years)?

3. In which country do you currently work?

4. In which setting do/did you mainly work?

Choose: private practice, regional hospital, academic or large tertiary hospital, other. If other: please specify.

5. Do you have the possibility to offer genetic testing for ADPKD in your unit?

Choose: yes, no.

6. Is your institute providing only laboratory services for ADPKD genetic testing?

Choose: yes, no. If yes, please stop here and send the survey.

7. Does your center offer the possibility for professional genetic counseling of ADPKD patients by a geneticist?

Choose: yes, no.

8. Are you a medically qualified specialist in genetics who has offered genetic counseling to patients in the last year?

Choose: yes, no.

9. Is (was) your center involved in patient- oriented ADPKD research?

Choose: yes, no.

10. Do you (strongly) disagree or (strongly) agree with the following statement?

Score from 1 (=strongly disagree) to 6 (=strongly agree).

- Health professionals should encourage clinical testing for ADPKD comorbidities (such as hypertension and proteinuria) in asymptomatic minor offspring from known ADPKD families.
- Health professionals should encourage clinical testing for ADPKD comorbidities (such as

hypertension and proteinuria) in asymptomatic adult offspring from known ADPKD families.

- Health professionals should encourage predictive genetic testing of ADPKD in asymptomatic minor offspring form known ADPKD families.

- Health professionals should encourage predictive genetic testing of ADPKD in asymptomatic adult offspring form known ADPKD families.

- Asymptomatic minor should encourage predictive genetic testing of ADPKD without the involvement and consent of his/her parents.

- Parents with known ADPKD have the right to decide if an asymptomatic minor should be clinically and/or genetically tested for ADPKD.

11. Who is responsible for ensuring that the minor is informed about his genetic risk for ADPKD at adulthood?

Choose: only the treating healthcare professional, mainly the treating healthcare professional, shared responsibility of treating healthcare professional and the parents, mainly the responsibility of the parents or only the responsibility of the parents.

12. You are asked for counseling a 35 year old ADPKD patient who has an asymptomatic child of 6 years old. Several affected family members are known with early disease manifestation. What would you recommend for this child? Several options are possible.

- Recommend annually blood pressure and urine checkup
- Recommend renal imaging testing
- Recommend genetic testing for child and parents
- Recommend clinical and/or genetic testing at adult age
- No investigations in childhood because no definite diagnosis method exists
- No investigations in childhood because the disease does not manifest before adulthood
- No investigations in childhood because there is no available treatment
- No investigations in childhood because of the risk of psychological stress

- No investigations in childhood because of possible insurance problems

13. Do you inform your patients about the possibility of prenatal genetic diagnosis for ADPKD by means of chorionic villus sampling or amniocentesis?

Choose: yes, all patients; yes, but only upon the patient's request or no.

14. Do you inform your ADPKD patients about the option of preimplantation genetic diagnosis?

Choose: yes, all patients; yes, but only upon the patient's request or no.

15. Do you (strongly) agree or (strongly) disagree with the following statements?

Score from 1 (=strongly disagree) to 6 (= strongly agree).

- Prenatal genetic diagnosis for ADPKD by means of chorionic villus sampling or amniocentesis is ethically justified?

- Termination of pregnancy for APKD is ethically justified?

- Preimplantation genetic diagnosis for ADPKD is ethically justified?

16. Comments are most welcome! (open field)
